# Revealing the air pollution burden associated with internal Migration in Peru

**DOI:** 10.1038/s41598-020-64043-y

**Published:** 2020-04-28

**Authors:** Gabriel Carrasco-Escobar, Lara Schwarz, J. Jaime Miranda, Tarik Benmarhnia

**Affiliations:** 1 Health Innovation Lab, Institute of Tropical Medicine “Alexander von Humboldt”, Universidad Peruana Cayetano Heredia, Lima, Peru; 2 Division of Infectious Diseases, Department of Medicine, University of California, San Diego, CA USA; 3 Department of Family Medicine and Public Health, University of California, San Diego, CA USA; 4 Scripps Institution of Oceanography, University of California, San Diego, CA USA; 5 CRONICAS Centre of Excellence in Chronic Diseases, Universidad Peruana Cayetano Heredia, Lima, Peru; 6 School of Medicine, Universidad Peruana Cayetano Heredia, Lima, Peru

**Keywords:** Environmental impact, Epidemiology

## Abstract

This study aims to quantify changes in outdoor (ambient) air pollution exposure from different migration patterns within Peru and quantify its effect on premature mortality. Data on ambient fine particulate matter (PM_2.5_) was obtained from the National Aeronautics and Space Administration (NASA). Census data was used to calculate rates of within-country migration at the district level. We calculated differences in PM_2.5_ exposure between “current” (2016–2017) and “origin” (2012) districts for each migration patterns. Using an exposure-response relationship for PM_2.5_ extracted from a meta-analysis, and mortality rates from the Peruvian Ministry of Health, we quantified premature mortality attributable to each migration pattern. Changes in outdoor PM_2.5_ exposure were observed between 2012 and 2016 with highest levels of PM_2.5_ in the Department of Lima. A strong spatial autocorrelation of outdoor PM_2.5_ values (Moran’s I = 0.847, p-value=0.001) was observed. In Greater Lima, rural-to-urban and urban-to-urban migrants experienced 10-fold increases in outdoor PM_2.5_ exposure in comparison with non-migrants. Changes in outdoor PM_2.5_ exposure due to migration drove 137.1 (95%CI: 93.2, 179.4) premature deaths related to air pollution, with rural-urban producing the highest risk of mortality from exposure to higher levels of ambient air pollution. Our results demonstrate that the rural-urban and urban-urban migrant groups have higher rates of air pollution-related deaths.

## Introduction

Migration is increasingly recognized as an important determinant for population health^1–4^. Nowadays, more than 244 million international migrants are estimated globally^5^ with considerable gaps in health care services. Furthermore, a significantly larger number of internal migrants − people moving within their country of birth − has been documented^4^, with a particular health burden despite being nationals. Migrants are commonly exposed to a range of challenges such as low quality of labor conditions, massive vehicular traffic, sedentarism (low level of physical activity), lack of social support, limited access to healthy food choices^6^, and stress^7,8^. Social vulnerability from economic instability, social isolation, poor access to healthcare services, increased exposure to infectious diseases, and traumatic events related to the migration itself have also been known to affect migrants’ health^9–14^.

In addition to these stressors, migrants are exposed to new environments, which may have further impacts. Environmental changes and exposures have been studied as drivers of migration^15–17^, including how exposure to hazards or availability of ecosystem services can lead residents’ to migrate away from these harmful environments. Although there is abundant literature studying how environmental hazards can drive emigration, only one study conducted in the United States (U.S.) has investigated how migrants may also be at a differential risk for environmental exposures. Interestingly, this study found that international immigrants in the U.S. are generally less exposed to chemical toxins, while specific groups such as Mexican immigrants in high income areas have a disproportionate exposure^18^. To our knowledge, no study has considered exposure to outdoor (ambient) air pollution as an environmental health risk of concern for migrant populations.

Exposure to outdoor air pollution is one of the leading environmental causes of mortality globally^19^. The burden of air pollutants is 6.4 million years of life lost^20^. Most health effects of air pollution have been documented in respiratory diseases, cardiovascular diseases, allergenic diseases, and diabetes, affecting individuals throughout the life course from developing fetuses to elderly populations^21^. Particulate matter under 2.5 micrometers (PM_2.5_) is known to be one of the more harmful pollutants due to its ability to penetrate deep into the lungs and enter the bloodstream^22^. With major increases in urbanization and migration to urban centers worldwide, which have higher levels of air pollution and other environmental exposures, the potential health implications of these changes and burden of migrant populations merits attention^23^. The study of health exposures among migrants also needs to further characterize the different profiles of within country migrants, as recently exemplified in the case of obesity^24^.

As a country with a high rate of internal migration and high levels of particulate matter in some regions, Peru is a unique context to study this topic. After a period of the political violence (armed internal conflict during the 1970–1990’s)^25^ with approximately 120,000 displaced families from the Andes to the Capital city of Lima^26^, nowadays most rural-to-urban migration has been largely driven by economic reasons, to seek access to better services. However, slow process of formal land titles and rising property prices in Lima have led some recent migrants to poor housing conditions in shantytowns with lack of water or electricity supply^27^. These harmful conditions expose migrants to environmental determinants that can lead to health disparities, such as increasing labor hours and unexpected health seeking costs that greatly affect low-income populations and reinforce the poverty cycle and negative health outcomes.

Previous studies have reported the air pollution status in Peru and highlighted the important geographical variability of air contaminants^28–31^. In addition, several studies have shown the impact of rural-to-urban migration in Peru on infectious and non-communicable diseases^32–43^ driven by changes in diet and physical activity patterns, increased poverty, and restricted access to health care. However, limited literature addresses outdoor air pollution effects in the migrant population, and to our knowledge, no study has quantified the health burden associated with changes in exposure to ambient air pollution due to migration between different locations.

In this study, we aimed to quantify changes in outdoor air pollution exposure related to different internal migration patterns (rural-to-rural, rural-to-urban, urban-to-rural, and urban-to-urban) in Peru, identify spatial clusters where high rates of migrants and high levels of PM_2.5_ collide, and finally quantify the associated changes in premature mortality using a health impact assessment methodology.

## Methods

### Study area and population

**Figure 1:**
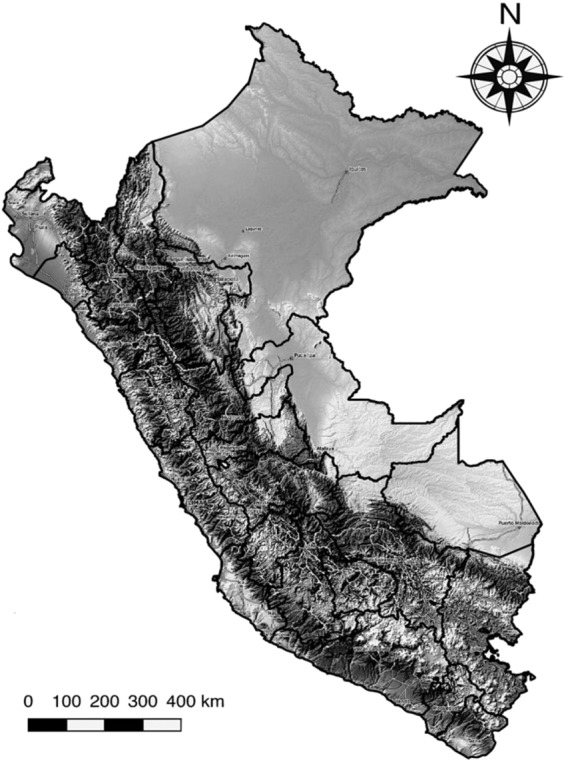
Study area. Contrasting ecological areas (Coast, Andes, and Jungle) in Peru. Solid lines represent the 25 Departments (administrative level 1). Map was produced using QGIS 3.6.3 (QGIS Development Team, 2019. QGIS Geographic Information System. Open Source Geospatial Foundation Project. http://www.qgis.osgeo.org/). Map tiles by OpenTopoMap (Kartendaten: © OpenStreetMap-Mitwirkende, SRTM | Kartendarstellung: © OpenTopoMap; http://www.opentopomap.org) under creative common licence CC BY-SA 3.0 (https://creativecommons.org/licenses/by-sa/3.0/).

This study was conducted in Peru for the 2012–2017 period. Administrative units in Peru are organized in 25 Departments (level 1), 196 provinces (level 2), and 1873 districts (level 3). Data encompasses information about 29,381,884 inhabitants in an area of 1,285,216 km^2^. Major ecological areas in the country were divided into the Coast, Andes, and Jungle (Fig. 1). This study used data at the smallest administrative level (districts), which were further classified as: districts of the capital (Greater Lima), big cities (population > 150,000), intermediate cities (pop. between 20,000–150,000), and small cities (pop. between <20,000).

### Data sources

#### Migration rate

Census data was provided by the National Institute of Statistics and Informatics (INEI in Spanish) via the REDATAM platform. Information from the 2017 Census was used to calculate within-country migration rates. In the census, all inhabitants were asked to report the district where they are currently living (2016–2017), henceforth referred to as “current district” and the district where they lived 5 years ago (2012), henceforth referred to as “origin district”. Children under 5 were excluded from this question. In addition, international migrants − people with a country of birth different than Peru − were excluded to analyze only within-country migration. After exclusion criteria, the information of 26,779,984 (91%) inhabitants was analyzed.

For this study, non-migrants were defined as population that reported the same origin and current district and migrants were defined as population for which the origin and current district differed. Origin and current districts were labeled as rural or urban according to the INEI National registry classification and rural-to-rural, rural-to-urban, urban-to-rural, and urban-to-urban migration status were constructed. The distribution of districts relative to rural/urban category and city type is presented in Supplementary Table 1.

For each of the 1873 districts the number of non-migrants, immigrants (with details on origin districts) and out-migrants (with details on destination districts) were computed. The migration rate was calculated as the number of migrants (immigrants or out-migrants independently) in each district divided by the total population in the same district. Standardized migration rates were calculated by multiplying the migration rate by the proportion of population in each district in 2017 (district population/country population). REDATAM exported data were formatted and processed using R software v.3.6.2 (R Core Team (2019). R: A language and environment for statistical computing. R Foundation for Statistical Computing, Vienna, Austria. URL https://www.R-project.org).

#### Air quality

Particulate matter with a diameter less than 2.5 micrometers, PM_2.5_, was used a measure of outdoor air pollution in this study given the strong evidence of its effects on adverse health outcomes^44,45^. Air quality data from the NASA - Socioeconomic Data and Applications Center (SEDAC) was used^46^. SEDAC provided an annual global surface of concentrations (micrograms per cubic meter - µg/m^3^) of mineral dust and sea-salt filtered PM_2.5_^46,47^. Although PM_2.5_ predictions using ground measurements and chemical transport models in addition to satellite data have been reported recently for Peru that are likely more accurate^48^, this data was only available for Lima (the capital) preventing comparisons between rural and urban settings. Previous studies^47^ demonstrated that SEDAC-NASA data were highly consistent (R^2^ = 0.81) with out-of-sample cross-validated PM_2.5_ concentrations from ground stations in the Global Burden of Disease (GBD) network^49^.

As a result, PM_2.5_ gridded data sets were provided at a spatial resolution of 0.01 degrees^46^. Most proximate datasets to the study period were used for the analysis, 2012 and 2016. We harmonized migration and air quality data at the same spatial scale, by estimating the median PM_2.5_ (SEDAC gridded data) at each district. Spatial boundaries of the 1873 districts were obtained from the National Institute of Geography (IGN in Spanish). Data was provided in shapefile format with tolerances of 1,000 m and 1,000,000 m^2^.

Data processing was conducted in Google Earth Engine (GEE)^50^. SEDAC dataset and IGN shapefiles were uploaded to GEE as assets. For data visualization purposes, districts were classified in 6 categories according the levels of PM_2.5_ in micrograms per cubic meter (<10, 10–15, 15–20, 20–25, 25–30, >30) relative to the WHO air quality guideline^51^.

#### Baseline mortality and exposure-response data

Mortality data (all causes) was collected at the departmental-level from the national center of epidemiology, prevention and control of disease of the Ministry of Health of Peru^52^. Each district was assigned the mortality rate for the department which it is nested.

The exposure-response relationship for PM_2.5_ was extracted from a review conducted by Hoek *et al*. (2013) which conducted a meta-analysis on cardio-respiratory mortality from air pollution exposure from epidemiological studies around the globe^53^. The overall estimate for all-cause mortality of 1.06 (95%CI: 1.04, 1.08) per 10 μg/m^3^ increase in PM_2.5_ exposure was used.

### **Spatial analysis between PM**_**2.5**_**and migration**

To determine the spatial dependence of PM_2.5_ concentration and its association with migration status, spatial autocorrelation analyses and a spatial Bayesian regression were conducted. First, to determine the spatial autocorrelation of PM_2.5_, meaning that the values of PM_2.5_ in each district is influenced by the values of PM_2.5_ on their neighborhood districts, a Global Moran’s *I* and Local Getis-Ord Gi* statistics were calculated using a first-order queen contiguity-based weighted neighborhood. The statistic under the null hypothesis of spatial randomness was calculated by randomly permuting the observed values over the locations. The z-distribution was computed based on spatial random data sets (the permuted data sets). No statistical testing was applied over the Gi* statistic to prevent bias due to multiple and dependent tests^54,55^. Clusters were categorized based on the Gi* statistic sign in high- (hotspot) or low- (coldspot) concentration areas and percentile (90%, 95%, 99%) of the z-distribution. Then the migration rates in each PM_2.5_ cluster were analyzed.

To assess the relation between migration status (independent variable) and ‘change in PM_2.5_ exposure’ defined as the difference in PM_2.5_ between 2012 and 2016 (dependent variable) accounting for the spatial structure of the districts in Peru, a Bayesian linear model was fitted using Integrated Nested Laplace Approximation (INLA, www.r-inla.org)^56^ for all districts in Peru (1873). A spatial model was formulated by including a spatial structure using a convolution prior that combined area-specific overdispersion and a neighborhood dependency structure^57,58^. See S1 supplementary methods for specification of prior and hyper-prior distributions.

### Quantifying attributable mortality

We used a Health Impact Assessment (HIA) methodology to quantify mortality attributable to change in PM_2.5_ caused by internal migration. HIAs have been used to quantify the mortality or morbidity associated with air pollution in various regions^59,60^ and are a standard tool to quantify the global burden of disease. These are based on exposure-response functions (ERF) for particulate matter obtained from epidemiological studies and can be extrapolated to a population to understand the burden and inform environmental policies^61^.

We first estimated changes in outdoor air pollution exposure due to migration by calculating the difference for each district to district migration (“current” and “origin”) in the year 2016. This represents the counterfactual scenario in which a migrant had not migrated and remained in the same district and therefore exposed to PM_2.5_ in the origin district.

We then calculated an attributable fraction (AF) using the risk ratio of exposure-response relationship extracted from meta-analysis of PM_2.5_ impacts, using the following equation [RR-1/RR]^53^. This AF was assumed to be the same for all districts in Peru and was multiplied by the PM_2.5_ concentration difference between the “current” and “origin” district and the all-cause mortality rate for each department in which the “current” district was nested to calculate the mortality rate attributable to change in PM_2.5_ exposure. The “current” department mortality rate was used for analysis to account for potential changes in baseline mortality between departments, to focus only on changes in outdoor air pollution exposure. For example, for those migrants that lived in Chachapoyas, Amazonas in 2012 and moved to Chorrillos, Lima in 2016, an ambient air pollution difference was considered at the district-level by considering the change in exposure between Chachapoyas and Chorrillos. This difference in ambient air pollution exposure was then multiplied by the mortality rate for the department of Lima which was considered homogenous and therefore assigned to the district of Chorrillos, and by the AF. This was considered the excess mortality for this migrant group, and differences were calculated for each migration pattern and weighted by the number of individuals that undertook this migration route to calculate average mortality rates from air pollution exposure attributable to migration. Finally, overall number of attributable deaths were calculated by taking into account the number of migrants that undertook each district-district route. 95% Confidence intervals were computed by calculating the attributable mortality based on variability of the PM_2.5_ ERF. All analyses were stratified by urbanicity of the origin and current district (rural-rural, urban-urban, rural-urban, urban-rural) and by department (using the migrants’ “current” department) to consider variation in risk spatially and by migration pattern.

## Results

### Spatial distribution of PM_2.5_

**Figure 2:**
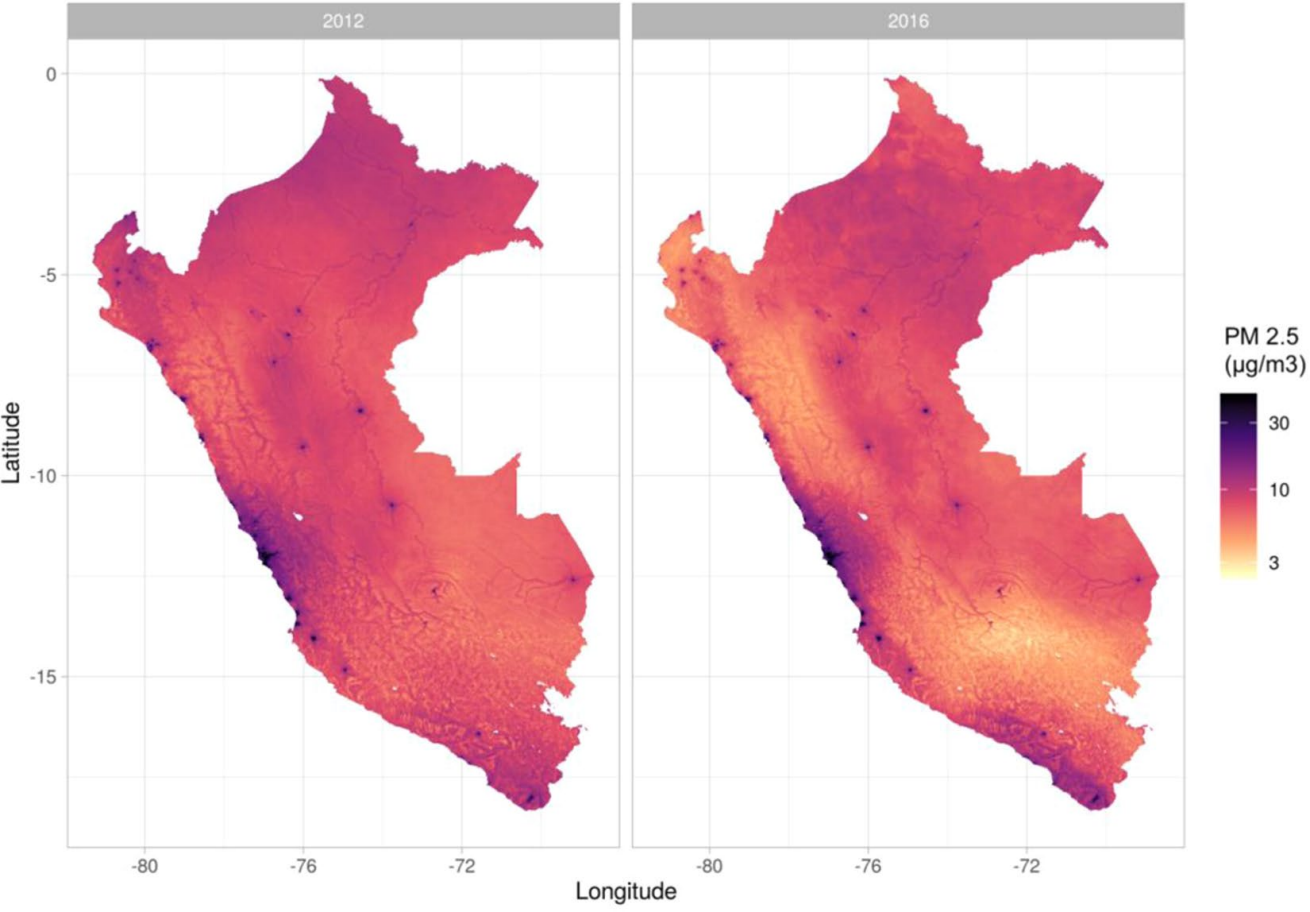
Country-wide spatial distribution of PM2.5 (μg/m^3^) at 0.01 degrees (~1.11 km). Estimates provided by NASA-SEDAC for 2012 (left) and 2016 (right). Maps were produced using R software v.3.6.2 (R Core Team (2019). R: A language and environment for statistical computing. R Foundation for Statistical Computing, Vienna, Austria. URL https://www.R-project.org/). based on NASA-SEDAC data (https://sedac.ciesin.columbia.edu/data/set/sdei-global-annual-gwr-pm2–5-modis-misr-seawifs-aod).

Important changes in PM_2.5_ were observed between 2012 and 2016 in the entire country (Fig. 2). Higher levels of PM_2.5_ were observed in the Department of Lima, southern coast, and in the Amazon Region (Fig. 2). The average PM_2.5_ levels were highly heterogeneous across city categories (Greater Lima, big, intermediate, and small cities, and rural areas). The highest average PM_2.5_ was located in Greater Lima with more than 4-folds higher in comparison with rural areas (Table 1).

**Table 1:** Migration rate and average PM_2.5_ in the rural-urban gradient in Peru.

Area	Number cities	Number districts	Population	Number of Migrants				Avg. PM_2.5_ (sd)
				urban-to-urban (%)	rural-to-urban (%)	urban-to-rural (%)	rural-to-rural (%)	
Greater Lima (Capital)	1	43	7,933,823	1,144,594 (14.43)	98,240 (1.24)	0 (0)	0 (0)	29.4 (9.57)
Big cities (pop > 150,000 inhab.)	9	12	2,669,156	253,222 (9.49)	62,524 (2.34)	0 (0)	0 (0)	15.2 (10.1)
Intermediate cities (pop. 20,000–150,000 inhab.)	88	185	9,116,366	967,790 (10.62)	210,306 (2.31)	25,283 (0.28)	11,972 (0.13)	10.4 (6.97)
Small cities (pop. <20,000)	195	1,633	7,060,639	306,702 (4.34)	75,687 (1.07)	139,093 (1.97)	60,256 (0.85)	6.76 (2.61)
Peru	196	1,873	26,779,984	2,672,308 (9.98)	446,757 (1.67)	164,376 (0.61)	72,228 (0.27)	7.69 (5.1)

### Spatial distribution of migration rates

**Figure 3:**
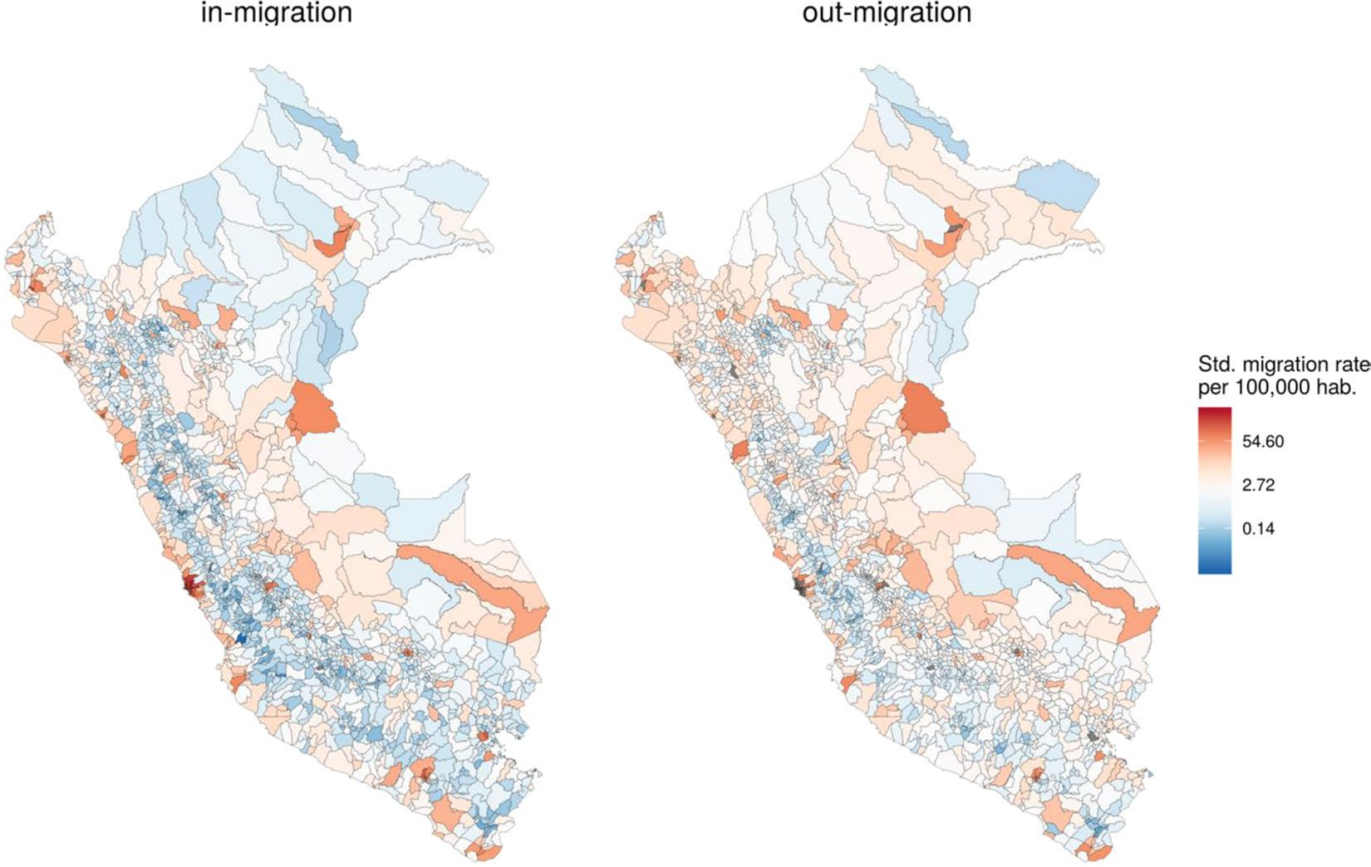
Spatial distribution of migration rates (2012–2016 period) at district-level in Peru. Standardized migration rates per 100,000 habitants for immigrants (left) and out-migrants (right). Maps were produced using R software v.3.6.2 (R Core Team (2019). R: A language and environment for statistical computing. R Foundation for Statistical Computing, Vienna, Austria. https://www.R-project.org/).

The district-level migration rate showed a scattered spatial pattern, however most migrants arrived to Greater Lima (Province of Lima) (Fig. 3 - left), which is also the city with the lowest out-migration in the country (Fig. 3 - right). The jungle and Andes are areas with low immigration and high out-migration rates, as opposed to what is observed in the coastal areas (Fig. 3). Interestingly, the 43 districts that make up Greater Lima received two-fold the migrant population (1,242,834 migrants) than small cities that make up 1,633 districts (581,738 migrants). The detailed distribution relative to the type of migration and city category is shown in Table 1. For rural-to-urban migrants, Greater Lima and Intermediate cities were the most important harboring places, and for urban-to-urban migrants as well.

### Change in PM_2.5_ exposure by migration status

**Figure 4:**
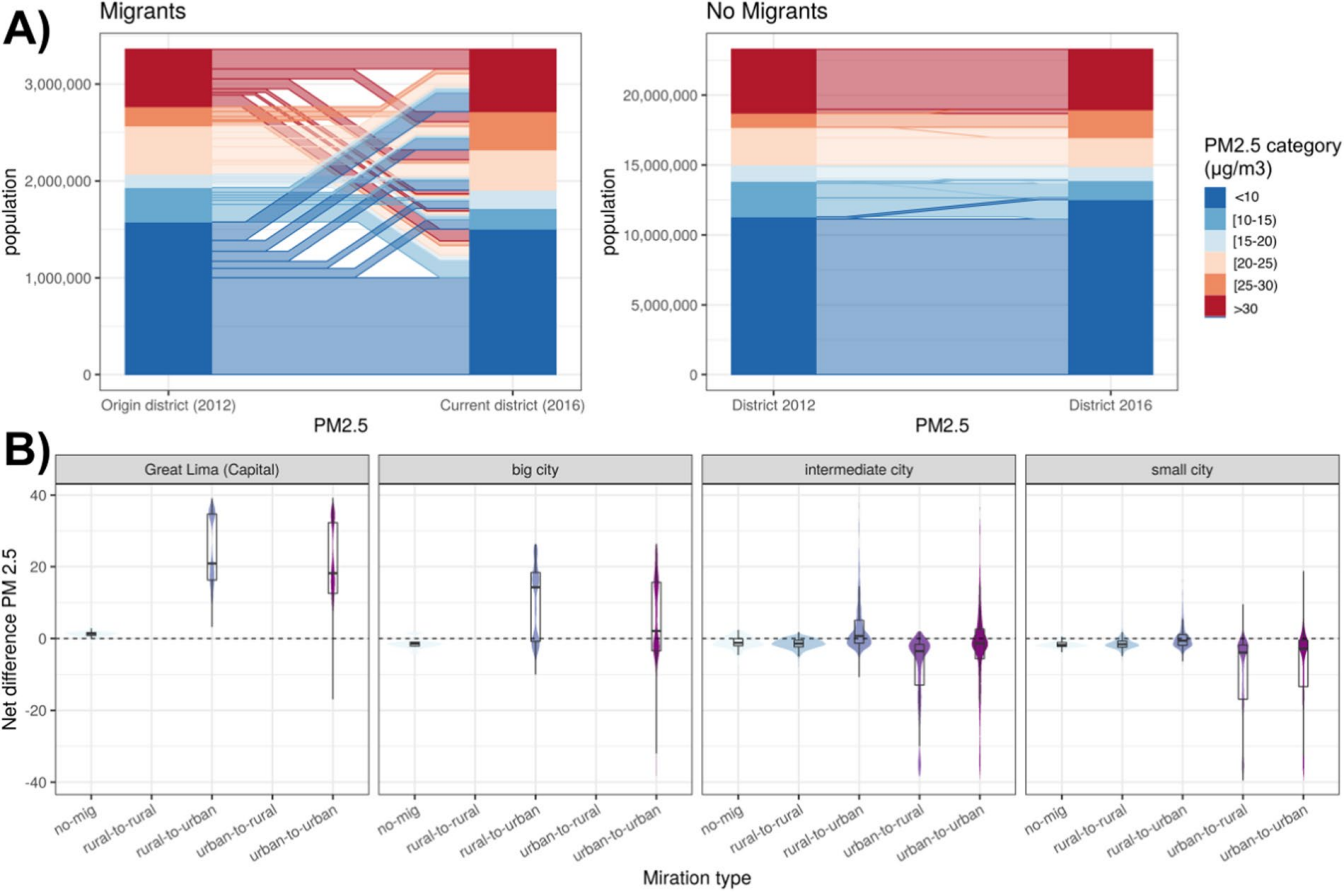
Exposure to PM_2.5_ in migrants and non-migrants in Peru. (**A**) Change in exposure to PM_2.5_ between origin and current districts of migrants, and between exposure in 2012 and 2016 in non-migrants. Districts were classified at 6 levels of exposure in micrograms per cubic meter (μg/m^3^) (dark blue: <10, blue: 10–15, light blue: 15–20, light orange: 20–25, orange: 25–30, red:>30). (**B**) Net difference in exposure to PM_2.5_ relative to type of migration (no migration, rural-to-rural, rural-to-urban, urban-to-rural, and urban-to-urban) and category of city (Greater Lima, big, intermediate and small cities). Figures were produced using R software v.3.6.2 (R Core Team (2019). R: A language and environment for statistical computing. R Foundation for Statistical Computing, Vienna, Austria. https://www.R-project.org/).

Larger changes in the PM_2.5_ exposure were observed in migration in comparison to non-migrants which only experienced a change in PM_2.5_ exposure due to environmental and social changes within the same district over time (Fig. 4A). Most (68%) of those exposed to high outdoor air pollution (PM_2.5_ > 30 μg/m^3^) were migrants from districts with a lower PM_2.5_ concentration. In Greater Lima, rural-to-urban and urban-to-urban migrants experienced 10-fold increases in PM_2.5_ exposure in comparison to non-migrants (Fig. 4B). Conversely, urban-to-rural and urban-to-urban migrants in small and intermediate cities were exposed to lower levels of PM_2.5_ in their receiving districts.

### Spatial analysis between PM_**2.5**_ and migration

**Figure 5:**
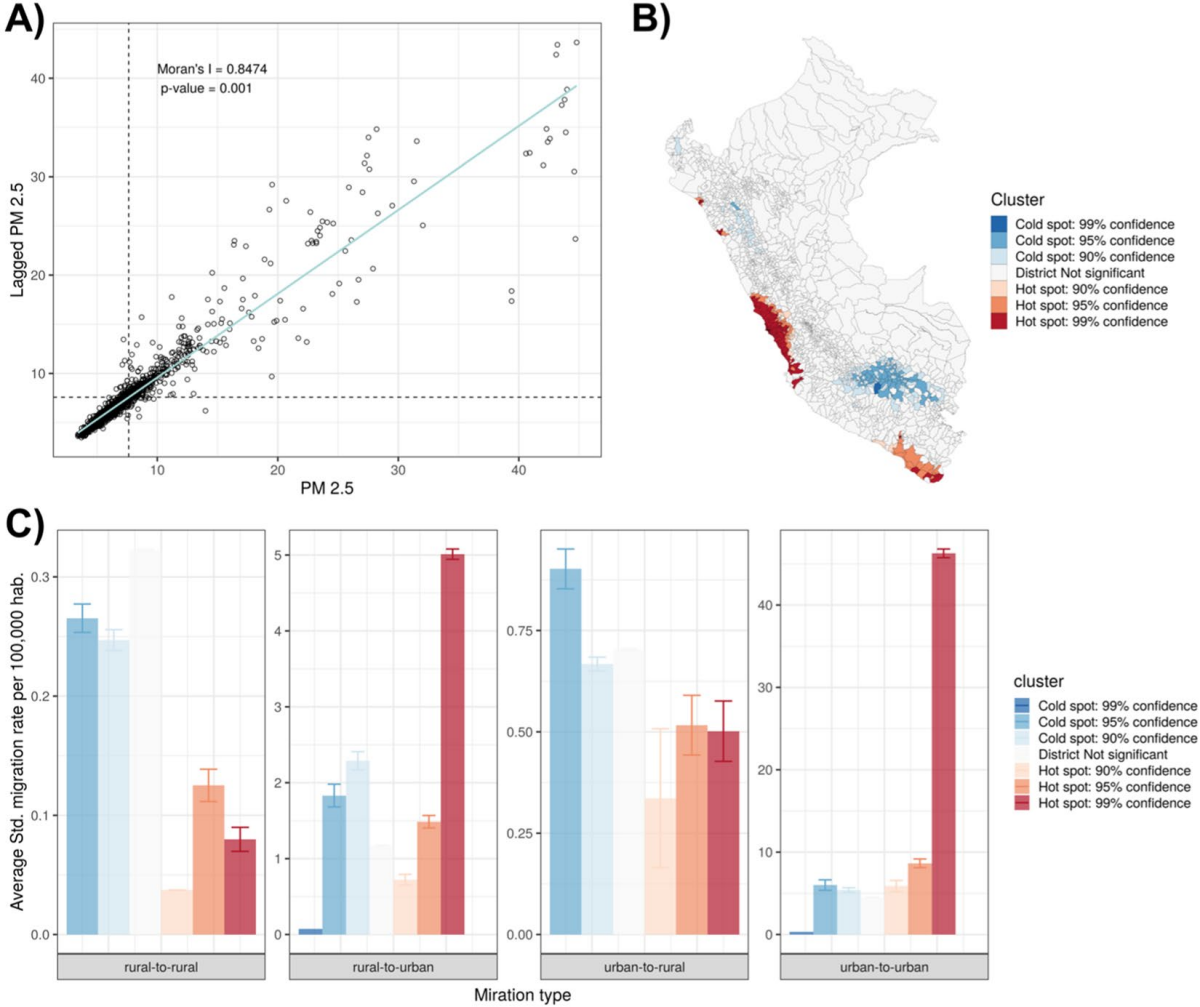
Spatial autocorrelation of PM_2.5_. (**A**) Global Moran’s *I* and. (**B**) Local Getis-Ord Gi* (right). (**C**) Standardized migration rates in clusters of PM_2.5_ relative to type of migration (no migration, rural-to-rural, rural-to-urban, urban-to-rural, and urban-to-urban). Clusters of PM_2.5_ were categorized based on the Gi* statistic sign in high- (hotspot) or low- (coldspot) concentration areas and percentile (90%, 95%, 99%) of the z-distribution. Maps and figures were produced using R software v.3.6.2 (R Core Team (2019). R: A language and environment for statistical computing. R Foundation for Statistical Computing, Vienna, Austria. https://www.R-project.org/).

An overall strong spatial autocorrelation of PM_2.5_ values (Moran’s I = 0.847, p-value=0.001) was observed in the study area (Fig. 5A). All detected hotspots clusters were located in the coastal areas of the country. As expected, the largest hotspot was located in the Province of Lima, however scattered hotspots in the southern and northern coast were also detected. Major coldspot clusters were located in the Andean Region (Fig. 5B). Overall, the average rural-to-urban and urban-to-urban migration rates was higher in PM_2.5_ hotspots than coldspots (Fig. 5C). Conversely, the average rural-to-rural and urban-to-rural migration rates was lower in PM_2.5_ hotspots than coldspots (Fig. 5C).

Using a Bayesian spatial model, we found that rural-to-urban migration patterns were associated with an increase in 9.88 µg/m^3^ (Credible Interval = 9.26, 10.51) of PM_2.5_ exposure when comparing exposure in the current to the origin district (Table 2). Conversely, notable reduction in PM_2.5_ exposure was observed for urban-to-rural migrants (Median = −8.36; 95% CI = −8.99: −7.74). Finally, moderate changes were observed for rural-to-rural and urban-to-urban migrants (Table 2).

**Table 2:** Parameter estimates of the spatial relation between change in PM_2.5_ exposure and migration status in Peru.

	Median	Std. Dev.	95% Credible Interval (CI)
***Intercept***	−1.484	0.308	[−2.089: −0.879]
***Migration status***			
Urban-to-urban	1.144	0.311	[0.534: 1.753]
Rural-to-urban	9.878	0.313	[9.263: 10.493]
Urban-to-rural	−8.351	0.316	[−8.971: −7.732]
Rural-to-rural	0.019	0.325	[−0.618: 0.657]

Bayesian linear model fitted with INLA accounting for the spatial structure of districts.

### Change in PM_2.5_ exposure and burden by migration status

**Figure 6:**
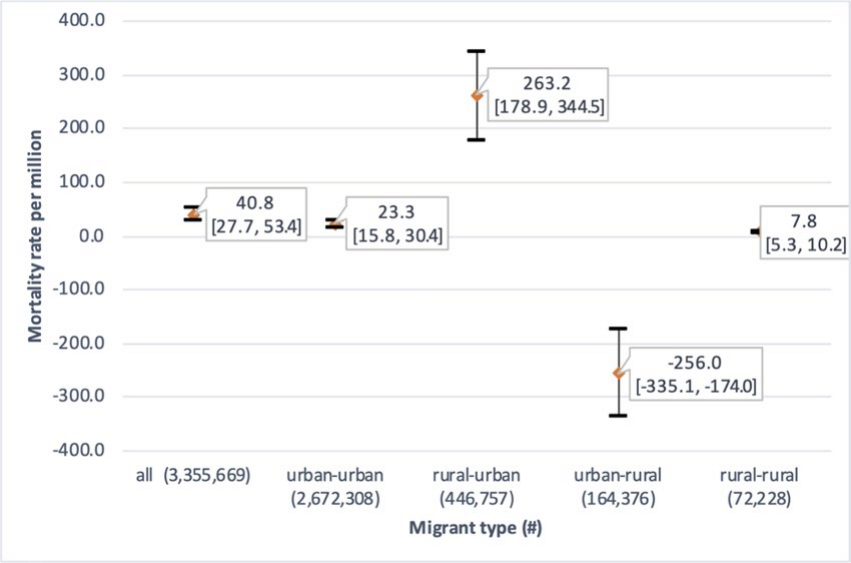
Graph showing difference in mortality rate (per million) and 95% confidence intervals related to air pollution exposure change from migration by migrant type.

Overall, we found that migration drove an additional 137 (95%CI: 93, 179) deaths related to outdoor air pollution exposure across Peru from 2012 to 2016. When considering only those that migrate from a rural-to-urban setting, there is an increase in 118 (95%CI: 80, 154) deaths related to outdoor air pollution. The largest migrant group, urban-to-urban, resulted in an increase in 62 (95%CI: 42, 81) ambient air pollution-related deaths, while the smallest migrant group, rural to rural, results in 0.5 (95%CI: 0.4, 0.7) additional deaths. There is a decrease in number of deaths for migrants going from an urban to rural setting, with 43 (95%CI: 29, 57) fewer deaths; this is one of the smaller groups, along with rural-rural migration. Mortality rates show similar results, with rural-urban revealing the greatest change in mortality rate and rural-rural migrants showing no change (Fig. 6). When considering the attributable deaths by migrants’ “current” department in 2016, it is apparent that migration to Lima is the main driver of outdoor air pollution related mortality from migration, where 313 deaths (95% CI: 213, 410) occur, while the majority of the other departments show decreases in outdoor air pollution-related mortality for migrants (Supplementary Table 2).

## Discussion

The substantial growth of internal human population mobility in low and middle income countries is changing population distributions across geographical areas, with subsequent changes in environmental exposures including air pollution, which fosters a need to study potential health implications in this setting. Migrants are a particularly vulnerable group in this context as they are moving to areas with higher or worse environmental risk profiles, often driven by economic, socio-political or geographical motives^62^. Besides changes in customs and risk behaviors, migrants also experience a change in environment. This study showed the first evidence of the relationship between changes in outdoor air pollution (PM_2.5_) exposure and burden related to migration status. Remarkable clustering patterns were observed in the spatial distribution of PM_2.5_ and migration rates. These distributions are spatially correlated in densely urban areas. This study highlights that rural-to-urban as well as urban-urban migrants experience a transition to environments with more deleterious ambient air pollution levels with a higher mortality burden, which adds to other previously reported socio-economic disadvantages. Additionally, although urban-to-urban migrants experience what may seem like a small change in exposure to particulate matter, this is associated with a considerable mortality burden, taking into account the important number of migrants that undertake this route. This demonstrates that even a slight increase in outdoor air pollution exposure associated with migration can have large impacts on population health, particularly when large groups are moving to environments with higher outdoor air pollution concentrations. The results of this study are important, because 1) it is the first study to quantify the effect of outdoor air pollution exposure on migrants health, and 2) it allows an understanding mobility and exposure patterns in Peru that can be integrated into urban planning policies to protect vulnerable populations.

Previous studies in China^63^ and the United States^64^ have argued that environmental inequalities may be shaped by social mechanisms such as residential segregation and racial income inequality. These same mechanisms were hypothesized in this study. In Greater Lima, the city that harbor most internal immigrants, the highest levels of PM_2.5_ are located in districts with the lowest average income. Albeit less marked than observed with PM_2.5_, districts with the highest migration rates are concurrently the most impoverished areas. Recent studies characterized the Peruvian internal migrants as primarily low-income population^24,65^, similar to other studies worldwide^66,67^. Taken together, the evidence points to the fact that the main mechanisms that drive migration and consequently higher PM_2.5_ exposure levels and burden are poverty and seeking of economic and educational opportunities. Many low-income rural populations are migrating to deleterious urban environments seeking better economic and educational opportunities but are exposed to much higher levels of PM_2.5_ and increased mortality risk. By identifying Lima as the department in Peru that far exceeds outdoor air pollution-related mortality burden for migrants, it can be considered a priority area for targeted ambient air pollution control measures, particularly for migrating populations.

Previous studies have described the effects of PM_2.5_ in non-communicable diseases (NCD), and respiratory diseases^68^. In Peru, increased risk of asthma^68,69^ and influenza^70^ were described in urban cities and migrant population, mediated by high values of PM_2.5_. Conditions such as soil organic matter, industry, and traffic have been suggested as drivers to the increased outdoor air pollution level which is attributed as a major determinant to the quality of life in the megacity of Lima^71^. As portrayed in the spatial analysis, hotspots of ambient air pollution are located on the coast of the country. Furthermore, these areas are located near main ports and heavily industrialized areas. These findings urge for new environmental health strategies to control these detrimental conditions. This study was able to exploit the different migration patterns and its associated health burden driven by changes in ambient air pollution exposure. Political commitment to address these issues will have a positive effect in reducing environmental-related health outcomes and the social and environmental gap observed in the migrant population.

This study is the first to describe a positive relation between internal migration and ambient air pollution exposure, and quantify the associated burden. Is important to mention that the social structure and social capital is also altered in the migrant population^72^. These conditions may deter the response capacity of the migrant population to affront environmental hazards for which recent migrants are exposed. However, it is important to acknowledge that migrants, and particularly those moving from rural to urban areas, may also experience increased economic opportunities and accessibility to healthcare. For example, financial reasons and other motivations related to improving people’s well-being is a strong driver of out-migration which may have a positive impact on the overall health of the migrant^62^. Although there are some nuances in the health effects of migration, it was beyond the scope of this study to consider these other factors, as the main purpose of this study was to quantify the outdoor air pollution-related health burden. Ultimately, it is well established that migrant populations in Peru are highly vulnerable to diseases due to environmental, social, and economic frailty^24,37^, and ambient air pollution exposure adds to this existing burden.

This manuscript focuses specifically on changes in outdoor ambient air pollution. However, indoor pollution from different sources such as cookstoves, is also a major health concern in Peru and globally^73–78^. Migration from rural to urban areas for example may have a beneficial effect on exposure to indoor air pollution due to changes in cooking fuels. Although we did not have access to such data, previous studies in Peru have found that indoor biomass stove can increase exposure to polycyclic aromatic hydrocarbons and particulate matter^74,79^. Additionally, while these are two different sources of exposure, interventions aimed at reducing residential wood burning have also been shown to reduce outdoor particulate matter concentrations showing important health benefits^80^.

Additional work could expand the exploration of other drivers of health and health changes, particularly for migrant populations, including other environmental exposures, potential positive changes in accessibility to healthcare and services as well as changes in PM_2.5_ exposure related to other internal migrant groups or external migration. However, in comparison to refugee migration, internal migration can be more precisely estimated^81^ due to the fact that mechanisms that influence internal migration follow long-term trends. However, the technical challenges to harmonize temporal and spatial data of internal displacements often prevents accurate quantification of internal migrants^1,67^. Furthermore, limited evidence showed the impact of internal migration on exposure to deleterious environments. The spatial autocorrelation analysis in this study has shown the large spatial effects in the relation between migration and PM_2.5_. These spatial effects might be due to social, economic, environmental (topological), or behavioral characteristics in the districts that receive the most migrants. This elucidates important evidence that geographically targeted interventions may have a great impact at the macro- and micro- scales.

Several limitations should be acknowledged in this study. First, migration estimates were computed based on whether the participant lived in the same district five years before. This cutoff is based on the census questionnaire and may represent a threat to consistency, due to the wide range from 5 years before to recent migrants (~1 day). Second, there is no question to check if participants are currently living in a permanent or temporary household, thus we cannot ascertain if they had a chronic exposure to these levels of outdoor air pollution. In the same way, migrants may arrive at a different place than where they were interviewed, and thus they may carry over the environmental exposures of all previous places where they had been settled that may yield to exposure misclassification. Third, as detected in the high-resolution images (Fig. 2 for reference), high variability of outdoor air pollution was observed within each district. Due to the lack of spatial resolution of migration status, ambient air pollution data was aggregated at the district level to be harmonized with migration data. This may yield biased conclusions due to PM_2.5_ and migration being non-stationary processes within districts. Further studies are suggested that account for fine-scale estimation of the relationship between migration and outdoor air pollution exposure. Also, despite residency exposure being a standard measure for ambient air pollution, this does not account for total exposure to air pollutants. This study did not include the exposure during work or leisure activities due to the lack of this data in the census. Additionally, we assumed a exposure-response relationship of PM_2.5_ based on existing literature on long-term effects of particulate matter based on studies primarily conducted in North America and Europe, while the true relationship may be different in the Peruvian context. However, the risk would be likely to be an underestimation as the majority of studies in the meta-analysis were from North America or Europe while impacts have been shown to be higher in Peru and other underrepresented countries^82^. Lastly, the baseline mortality rate considered for calculating attributable mortality risk was available only at the departmental level, therefore we had to assume homogeneity in mortality rates for districts within each department.

## Conclusion

This study was able to demonstrate the effect of migration on change in exposure to air pollution and quantify the mortality burden from this change. The clustered co-occurrence of high values of migration and PM_2.5_ suggest this relation might be shaped also by social and economic inequalities. Results suggest that internal migration in Peru drives an increase in mortality from air pollution exposure, with rural-urban migrants as one of the most vulnerable migrant groups, as well as the most prevalent. The findings of this study may represent the first steps to tailor strategies to improve the health of vulnerable populations such as internal migrants. Additionally, our results demonstrate the importance of considering air pollution as an environmental risk factors when studying migrant health in Peru and beyond.

## Supplementary information

Supplementary Information.

## Data Availability

Census data was provided by the National Institute of Statistics and Informatics (INEI in Spanish) via the REDATAM platform available in http://censos2017.inei.gob.pe/redatam/. Air pollution data (PM_2.5_) was provided by the NASA - Socioeconomic Data and Applications Center (SEDAC) available in https://sedac.ciesin.columbia.edu/data/set/sdei-global-annual-gwr-pm2–5-modis-misr-seawifs-aod.
