# Author Correction: Revealing the air pollution burden associated with internal Migration in Peru

**DOI:** 10.1038/s41598-020-77239-z

**Published:** 2020-11-24

**Authors:** Gabriel Carrasco-Escobar, Lara Schwarz, J. Jaime Miranda, Tarik Benmarhnia

**Affiliations:** 1 Health Innovation Lab, Institute of Tropical Medicine “Alexander Von Humboldt”, Universidad Peruana Cayetano Heredia, Lima, Peru; 2 Division of Infectious Diseases, Department of Medicine, University of California, San Diego, CA USA; 3 Department of Family Medicine and Public Health, University of California, San Diego, CA USA; 4 Scripps Institution of Oceanography, University of California, San Diego, CA USA; 5 CRONICAS Centre of Excellence in Chronic Diseases, Universidad Peruana Cayetano Heredia, Lima, Peru; 6 School of Medicine, Universidad Peruana Cayetano Heredia, Lima, Peru

Correction to: *Scientific Reports* 10.1038/s41598-020-64043-y, published online 28 April 2020

The original version of this Article contained errors due to a typo in the analytical code, which produced incorrect estimates and confidence intervals.

In the Abstract,

“Changes in outdoor PM_2.5_ exposure due to migration drove 137.1 (95% CI: 93.2, 179.4) premature deaths related to air pollution, with rural-urban producing the highest risk of mortality from exposure to higher levels of ambient air pollution.”

now reads:

“Changes in outdoor PM_2.5_ exposure due to migration drove 185 (95% CI: 2.7, 360) premature deaths related to air pollution, with rural-urban producing the highest risk of mortality from exposure to higher levels of ambient air pollution.”

In the Methods section, under subheading ‘Quantifying attributable mortality’,

“We first estimated changes in outdoor air pollution exposure due to migration by calculating the difference for each district to district migration (“current” and “origin”) in the year 2016.”

now reads:

“We first estimated changes in outdoor air pollution exposure due to migration by calculating the difference for each district to district migration (“current” and “origin”) in the year 2016 after truncating the PM_2.5_ estimates for the "current" and "origin" districts at the 5th/95th percentile.”

and,

“We then calculated an attributable fraction (AF) using the risk ratio of exposure-response relationship extracted from meta-analysis of PM_2.5_ impacts, using the following equation [RR-1/RR]^53^. This AF was assumed to be the same for all districts in Peru and was multiplied by the PM_2.5_ concentration difference between the “current” and “origin” district and the all-cause mortality rate for each department in which the “current” district was nested to calculate the mortality rate attributable to change in PM_2.5_ exposure.”

now reads:

“We calculated a relative risk of the exposure by multiplying the change in PM_2.5_ between "current" and "origin" district by the ln(ERF) and exponentiating this product. An attributable fraction (AF) was then estimated applying the following equation to the relative risk of the exposure [RR-1/RR]. This AF was then multiplied by the all-cause mortality rate for each department in which the “current” district was nested to calculate the mortality rate attributable to change in PM_2.5_ exposure.”

and,

“This difference in ambient air pollution exposure was then multiplied by the mortality rate for the department of Lima which was considered homogenous and therefore assigned to the district of Chorrillos, and by the AF.”

now reads:

“This difference in ambient air pollution exposure was then used to calculate attributable fraction which was multiplied by the mortality rate for the department of Lima which was considered homogenous and therefore assigned to the district of Chorrillos.”

In the Results section, under subheading ‘Change in PM_2.5_ exposure and burden by migration status’,

“Overall, we found that migration drove an additional 137 (95% CI: 93, 179) deaths related to outdoor air pollution exposure across Peru from 2012 to 2016. When considering only those that migrate from a rural-to-urban setting, there is an increase in 118 (95% CI: 80, 154) deaths related to outdoor air pollution. The largest migrant group, urban-to-urban, resulted in an increase in 62 (95% CI: 42, 81) ambient air pollution-related deaths, while the smallest migrant group, rural to rural, results in 0.5 (95% CI: 0.4, 0.7) additional deaths. There is a decrease in number of deaths for migrants going from an urban to rural setting, with 43 (95% CI: 29, 57) fewer deaths; this is one of the smaller groups, along with rural-rural migration. Mortality rates show similar results, with rural–urban revealing the greatest change in mortality rate and rural-rural migrants showing no change (Fig. 6). When considering the attributable deaths by migrants’ “current” department in 2016, it is apparent that migration to Lima is the main driver of outdoor air pollution related mortality from migration, where 313 deaths (95% CI: 213, 410) occur, while the majority of the other departments show decreases in outdoor air pollution-related mortality for migrants (Supplementary Table 2).”

now reads:

“Overall, we found that migration drove an additional 185 (95% CI: 2.7, 360) deaths related to outdoor air pollution exposure across Peru from 2012 to 2016. When considering only those that migrate from a rural-to-urban setting, there is an increase in 87 (95% CI: 59, 114) deaths related to outdoor air pollution. The largest migrant group, urban-to-urban, resulted in an increase in 120 (95% CI: −24, 259) ambient air pollution-related deaths, the smallest migrant group, rural to rural, results in 0.56 (0.01, 1.1) additional deaths. There is a decrease in number of deaths for migrants going from an urban to rural setting, with −23 (95% CI: −32, −14) fewer deaths; this is one of the smaller groups, along with rural-rural migration. Mortality rates show similar results, with rural-urban revealing the greatest change in mortality rate and rural-rural migrants showing little change (Fig. 6). When considering the attributable deaths by migrants’ “current” department in 2016, it is apparent that migration to Lima is the main driver of outdoor air pollution related mortality from migration, where 229 deaths (95% CI: 143, 311) occur, while the majority of the other departments show decreases in outdoor air pollution-related mortality for migrants (Supplementary Table 2).”

In the Discussion section,

“This study highlights that rural-to-urban as well as urban-urban migrants experience a transition to environments with more deleterious ambient air pollution levels with a higher mortality burden, which adds to other previously reported socio-economic disadvantages. Additionally, although urban-to-urban migrants experience what may seem like a small change in exposure to particulate matter, this is associated with a considerable mortality burden, taking into account the important number of migrants that undertake this route.”

now reads:

“This study highlights that rural-to-urban migrants experience a transition to environments with more deleterious ambient air pollution levels with a higher mortality burden, which adds to other previously reported socio-economic disadvantages.”

Additionally, this error generated incorrect graph data for Figure 6.

Finally, the amended health impact assessment resulted in revised estimates in the Supplementary Information file.

These errors have now been corrected in the PDF and HTML versions of this article, and the Supplementary Information has been updated.

