# Supplementary material for: Revealing the air pollution burden associated with internal Migration in Peru
Source: Sci Rep. 2020 Apr 28;10:7147. doi: 10.1038/s41598-020-64043-y (PMC7188878; doi:10.1038/s41598-020-64043-y)
Supplement: Supplementary file 1 — Supplementary Information. [file 41598_2020_64043_MOESM1_ESM.docx]

**Revealing the air pollution burden associated with internal Migration in Peru**

Gabriel Carrasco-Escobar^1,2,^*, Lara Schwarz^3,4^, J. Jaime Miranda^5,6^, Tarik Benmarhnia^3,4^

^1^ Health Innovation Lab, Institute of Tropical Medicine “Alexander von Humboldt”, Universidad Peruana Cayetano Heredia, Lima, Peru

^2^ Division of Infectious Diseases, Department of Medicine, University of California, San Diego, CA, USA

^3^ Department of Family Medicine and Public Health, University of California, San Diego, CA, USA

^4^ Scripps Institution of Oceanography, University of California, San Diego, CA, USA

^5^ CRONICAS, Centre of Excellence in Chronic Diseases, Universidad Peruana Cayetano Heredia, Lima, Peru.

^6^ School of Medicine, Universidad Peruana Cayetano Heredia, Lima, Peru

*** Corresponding Author:** Gabriel Carrasco-Escobar, MS, PhD(c) ([gabriel.carrasco@upch.pe](mailto:gabriel.carrasco@upch.pe))

**Keywords:** Air pollution, internal migration

**SUPPLEMENTARY INFORMATION**

1. **SUPPLEMENTARY METHODS**

**Prior and hyperprior distribution specification**

Model parameters were estimated in a Bayesian framework, using Integrated Nested Laplace Approximation (INLA, [www.r-inla.org](http://www.r-inla.org)) in R version 3.6.0. Parameter uncertainty is accounted for by assigning prior distributions to the parameters. The spatial component of the model was included using the BYM-model (Besag-York-Mollier) that includes a spatially unstructured ($\varphi_{s})$, and structured ($\upsilon_{s})$random effects using a convolution prior that combined area-specific overdispersion and a neighborhood dependency structure ^1,2^. The hyperparameters are the precision τ_1_ of the iid model ($\varphi_{s}$) and the precision τ_2_ of the besag model ($\upsilon_{s}$). The precision parameters are represented as

$$\theta=\left( \theta_{1},\theta_{2} \right)=(\log\tau_{1},\log\tau_{2})$$

and the prior is defined on θ. The hyperparameter specification for $\tau_{1}$ and $\tau_{2}$ was prior=loggamma, param=1 5e-04, initial=4.

**References**

1. Besag, J., Green, P., Higdon, D. & Mengersen, K. Bayesian Computation and Stochastic Systems. *Stat. Sci.* **10**, 3–41 (1995).

2. Lowe, R. *et al.* The development of an early warning system for climate-sensitive disease risk with a focus on dengue epidemics in Southeast Brazil. *Stat. Med.* **32**, 864–883 (2013).

1. **SUPPLEMENTARY TABLE 1.** Distribution of districts relative to rural/urban category and city type.

|  | **Great Lima (Capital)** | **Big city** | **Intermediate city** | **Small city** | **Total** |
| --- | --- | --- | --- | --- | --- |
| Rural | 0 | 0 | 25 | 868 | 893 |
| Urban | 43 | 12 | 160 | 765 | 980 |
| **Total** | 43 | 12 | 185 | 1633 | 1873 |

**SUPPLEMENTARY TABLE 2.** Attributable mortality to air pollution exposure change related to migration by their “current” department of residence (2016) Peru, 2012-2016.

| Department | Attributable mortality | 95% CI | |
| --- | --- | --- | --- |
| Amazonas | -6.30 | -8.24 | -4.28 |
| Ancash | -15.94 | -20.86 | -10.83 |
| Apurimac | -11.06 | -14.47 | -7.52 |
| Arequipa | -8.26 | -10.81 | -5.61 |
| Ayacucho | -12.26 | -16.04 | -8.33 |
| Cajamarca | -13.72 | -17.95 | -9.32 |
| Callao | 16.86 | 11.46 | 22.07 |
| Cusco | -23.95 | -31.34 | -16.27 |
| Huancavelica | -3.60 | -4.71 | -2.44 |
| Huanuco | -12.43 | -16.26 | -8.44 |
| Ica | -5.13 | -6.71 | -3.48 |
| Junin | -26.17 | -34.25 | -17.78 |
| La Libertad | 9.59 | 6.51 | 12.55 |
| Lambayeque | -1.06 | -1.39 | -0.72 |
| Lima | 313.30 | 212.87 | 409.97 |
| Loreto | -6.24 | -8.17 | -4.24 |
| Madre de Dios | -1.60 | -2.09 | -1.08 |
| Moquegua | -1.07 | -1.40 | -0.73 |
| Pasco | -4.16 | -5.45 | -2.83 |
| Piura | -19.07 | -24.96 | -12.96 |
| Puno | -9.03 | -11.82 | -6.13 |
| San Martin | -10.82 | -14.16 | -7.35 |
| Tacna | 0.60 | 0.41 | 0.79 |
| Tumbes | -3.10 | -4.04 | -2.10 |
| Ucayali | -8.30 | -10.83 | -5.62 |
